# Gene expression profiles of angiogenesis markers and microRNA-128 from the secretome of umbilical cord mesenchymal stem cells from *Macaca fascicularis*

**DOI:** 10.14202/vetworld.2025.558-564

**Published:** 2025-03-09

**Authors:** Hieronimus Adiyoga Nareswara Utama, Sela Septima Mariya, Ratih Rinendyaputri, Alvian Dumingan, Yoggi Ramadhani Purwaningtyas, Putri Retno Intan, Gita Iftitah Renitia, Hasta Handayani Idrus, Wireni Ayuningtyas, Rachmawati Noverina, Fathul Huda, Ahmad Faried, Sunarno Sunarno, Amarila Malik

**Affiliations:** 1 Graduate School of Pharmaceutical Sciences, Faculty of Pharmacy, Universitas Indonesia, Depok 16424, West Java, Indonesia; 2 Center for Biomedical Research, National Research and Innovation Agency (BRIN), Cibinong Science Centre, Jalan Raya Bogor KM 46, Cibinong, West Java, Indonesia 16911; 3 Bio Farma Stem Cell Research and Development, Bandung, Indonesia 40161; 4 Department of Neurology, Faculty of Medicine, Dr. Hasan Sadikin Central General Hospital/Universitas Padjadjaran, Bandung, Indonesia 40161; 5 Department of Neurosurgery, Faculty of Medicine, Universitas Padjadjaran, Bandung, Indonesia; 6 Division of Pharmaceutical Microbiology and Biotechnology, Faculty of Pharmacy, Universitas Indonesia, Depok 16424, West Java, Indonesia

**Keywords:** angiogenesis, hypoxic preconditioning, ischemic stroke, mesenchymal stem cells, microRNA-128, secretome

## Abstract

**Background and Aim:**

Angiogenesis and anti-apoptosis play crucial roles in ischemic stroke recovery. The mesenchymal stem cell (MSC) secretome, rich in bioactive molecules, presents a promising therapeutic avenue. However, optimizing the culture conditions to enhance the expression of angiogenic markers remains a challenge. This study examines the effect of hypoxic preconditioning on the expression of vascular endothelial growth factor (VEGF), monocyte chemoattractant protein-1 (MCP-1), matrix metalloproteinase-2 (MMP-2), and microRNA (miRNA-128) in the secretome of umbilical cord-derived MSCs (UC-MSCs) from *Macaca fascicularis*.

**Materials and Methods:**

UC-MSCs were cultured under normoxic (21% O_2_) and hypoxic conditions (1%, 3%, and 5% O_2_) for 48 h. The secretome was isolated, and reverse transcription-quantitative polymerase chain reaction was used to quantify the expression of VEGF, MCP-1, MMP-2, and miRNA-128. Expression levels were normalized to housekeeping genes and analyzed using statistical methods to determine significant differences among groups.

**Results:**

Hypoxic preconditioning significantly upregulated *VEGF* (1% O_2_), *MCP-1 (*5% O_2_), and *miRNA-128 (*5% O_2_) expression compared to normoxic conditions. Conversely, *MMP-2* expression was highest in normoxic conditions and downregulated under hypoxia. In addition, *miRNA-128* was found to be predominantly secreted into the extracellular space under hypoxic conditions rather than retained within cells.

**Conclusion:**

Hypoxic preconditioning effectively modulates the expression of key angiogenesis and anti-apoptotic markers in UC-MSCs. The study highlights the importance of optimizing oxygen levels to enhance the therapeutic potential of MSC-derived secretomes for ischemic stroke treatment. Future research should focus on *in vivo* validation and clinical translation of these findings.

## INTRODUCTION

The secretome is a set of biological factors secreted by stem cells [1], also known as the conditioned medium (CM). Secretome-based therapy is a new approach to restoring damaged tissues because it has many advantages over stem cells, such as hindering immune compatibility and infection issues [2]. It has been shown that secretomes are a promising treatment for various diseases, including ischemic stroke. However, standardized production and validation methods are required before they can be used [3]. Examining the molecular factors involved in stroke recovery is essential to understand the mechanism underlying secretome-based therapy.

In the process of recovery after a stroke, some biomolecules are involved at the molecular level, such as angiogenesis, which involves the creation of new blood vessels. This process is regulated by vascular endothelial growth factor (VEGF), which directly acts on neural progenitor cells to exert neurogenic effects [4, 5]. In addition to angiogenesis, the effectiveness of stem cell therapy is influenced by cell migration. Some proteins are involved in stem cell migration [5]. Monocyte chemoattractant protein-1 (MCP-1), a chemokine, plays a key role in macrophage and neuroblast migration to the infarct area [6, 7]. Another protein, matrix metalloproteinase-2 (MMP-2), is also involved in stem cell migration and neurogenesis, indicating its potential use as a marker in neuron cell regeneration therapy [8, 9]. After a stroke attack, apoptotic or anti-apoptotic proteins are simultaneously overexpressed and involved in some signaling pathways [10]. MicroRNA (miRNA), a small non-coding RNA that can regulate protein expression [11], can be used to inhibit apoptosis. One type of miRNA that can inhibit apoptosis is miRNA-128, which is also widely expressed in the brain [12]. miRNA-128-5p is a mature form of miRNA-128 with a forward orientation. It has been shown that miRNA-128-5p, which acts as an anti-apoptotic agent, can negatively regulate the DNA damage-inducible gene 45 gamma (Gadd45g) [13]. As molecules that are involved in the process of angiogenesis and anti-apoptosis, VEGF, MCP-1, MMP-2, and miRNA-128 have potential as markers for stroke therapy using secretomes.

To study the effect of stem cell preconditioning on the expression profiles of these markers at a low cost, we used non-human primates (NHP), which are frequently used in biomedical research, that is, long-tailed monkeys (*Macaca fascicularis*). Our previous study successfully cultured and characterized the phenotype and differentiation ability of cord-derived mesenchymal stem cells (UC-MSCs) obtained from NHP UC-MSCs [14]. It was reported that hypoxia treatment (1%, 3%, and 5% O_2_) induced the secretion of brain-derived neurotrophic factor and stromal cell-derived factor-1. This study aimed to evaluate the messenger RNA (mRNA) expression profiles of *VEGF*, *MCP-1*, and *MMP-2*. This study also examines epigenetic factors like *miRNA-128* from normoxic and hypoxic secretomes that were previously obtained by Dumingan *et al*. [14]. These profiles are essential for predicting the potential of the NHP UC-MSC secretome as a candidate for stroke therapy.

## MATERIALS AND METHODS

### Ethical approval

The study was conducted following animal welfare principles and has received ethical approval from the Biofarma Commission for Laboratory Animal Welfare and Use under ethical clearance number 01/IACUC_BF/X/22.

### Study period and location

This study was conducted from July to December 2023 at the Center for Primate Animal Studies (PSSP) of IPB, Bogor, for stem cell isolation activities; Laboratory of Infectious Disease Research, Ministry of Health, Jakarta, for stem cell preconditioning and secretome isolation activity; and Genomic Laboratory, KST Soekarno, National Research and Innovation Agency, Cibinong, for RNA extraction, complementary DNA (cDNA) synthesis, and reverse transcription polymerase chain reaction (RT-PCR) reactions.

### Sample collection

We used archived samples of secretome-producing NHP UC-MSC samples and their CM from our previous study by Dumingan *et al*. [14]. The umbilical cords of long-tailed monkeys were obtained from PT. Biofarma (Bandung, Indonesia).

Stem cells were incubated for 48 h in a two-gas incubator (Thermo Scientific, USA). Incubation conditions included normoxic (21% O_2_) and hypoxic environments with oxygen concentrations of 1%, 3%, and 5%. The secretome was separated by centrifugation at 161× *g* for 10 min at 4°C and filtered using a 0.22 μm syringe filter. The medium and cells were stored at −20°C before being used for further analysis.

### mRNA expression level profiles

#### Extraction of mRNA and cDNA synthesis

mRNA was extracted from stem cells using an RNA Simple Total RNA kit (Tiangen, China) following the manufacturer’s instructions. The extracted RNA was diluted to a final concentration of 3 ng/μL before cDNA synthesis using ReverTra Ace-α Kit (Toyobo, Japan) according to the manufacturer’s instructions. The reaction mixture was incubated at 30°C for 10 min, followed by incubation at 42°C for 20 min, and then heated at 95°C for 5 min. The mixture was then cooled down at 4°C for 2 min. The cDNA products can then be stored at −20°C for downstream analyses.

#### RT-quantitative PCR (RT-qPCR) for the mRNA expression level

RT-qPCR was performed using the SsoFast EvaGreen Supermix Kit (Bio-Rad, USA) according to the manufacturer’s protocol. The reactions began with initial enzyme activation at 95°C for 30 s, followed by 40 cycles of denaturation at 98°C for 5 s and annealing at 57°C for 15 s. All reactions were performed using a 7500 RT-PCR System (Applied Biosystem, USA).

### Epigenetic miRNA-128 profile

#### Extraction of miRNA-128 and cDNA synthesis

miRNA-128 was extracted from stem cells and secretomes using a miRcute miRNA Isolation Kit (Tiangen) according to the manufacturer’s protocol. After dilution to reach a concentration of 3 ng/μL, cDNA was synthesized using miRcute Plus miRNA First-strand cDNA Kit (Tiangen) according to the manufacturer’s protocol. The reactions were performed at 42°C for 60 min for adenine tailing and reverse transcription, followed by enzyme inactivation at 95°C for 3 min. The cDNA products can be stored at −20°C for downstream analyses.

#### miRNA expression levels using RT-qPCR

RT-qPCR was performed using a miRcute Plus miRNA qPCR Kit (Tiangen) according to the manufacturer’s protocol. The PCR reactions were initiated by the pre-denaturation stage at 95°C for 15 min, followed by the denaturation stage at 94°C for 20 s and the extension stage at 60°C for 34 s. The reactions were cycled 40 times using a 7500 RT-PCR System (Applied Biosystem). The oligonucleotide PCR primers used in this study are listed in Table 1 [15–20]:

**Table 1:** Oligonucleotide primers used for PCR reactions.

No.	Primer name	Primer sequence (5’➔ 3’)	Reference
1.	hsa-miR-128-1-5p	CGGGGCCGTAGCACTGTCTGAGA	[15]
2.	VEGF Forward	CTACTGCCATCCAATCGAGAC	[16]
	VEGF Reverse	GATCCGCATAATCTGCATGG	
3.	MCP-1 Forward	GCTGTGATCTTCAAGACCATTGTG	[17]
	MCP-1 Reverse	TGGAATCCTGAACCCACTTCTG	
4.	MMP-2 Forward	CATACAAAGGGATTGCCAGGA	[18]
	MMP-2 Reverse	GGTATTGCACTGCCAACTCT	
5.	U6 Forward	CTCGCTTCGGCAGCACA	[19]
	U6 Reverse	AACGCTTCACGAATTTGCGT	
6.	ACTB Forward	ACAGAGCCTCGCCTTTGC	[20]
	ACTB Reverse	CACGATGGAGGGGAAGAC	

PCR=Polymerase chain reaction, VEGF=Vascular endothelial growth factor, MCP=Monocyte chemoattractant protein, MMP=Matrix metalloproteinase, ACTB=β-actin

### Statistical analysis

All statistical analyses were performed using IBM Statistical Package for the Social Sciences Statistics for Windows, Version 23.0 (IBM Corp., Armonk, NY, USA). The relative gene expression levels of *VEGF*, *MCP-1*, *MMP-2*, and *miRNA-128* were quantified using the 2^−ΔCt^ method [21], with β-actin and U6 small nuclear RNA (*U6*) serving as reference genes for mRNA and miRNA normalization, respectively.

Comparisons between groups were conducted using one-way analysis of variance, followed by *post hoc* Tukey’s test for pairwise comparisons. When the data did not meet normality or homogeneity of variance assumptions (assessed through the Shapiro–Wilk test and Levene’s test, respectively), the Kruskal–Wallis test was used as a non-parametric alternative, followed by the Mann–Whitney U-test for *post hoc* pairwise comparisons.

For comparisons between normoxic and individual hypoxic groups, an independent samples t-test was performed. A p-value < 0.05 was considered statistically significant.

## RESULTS

### mRNA expression profile

**Figure 1:**
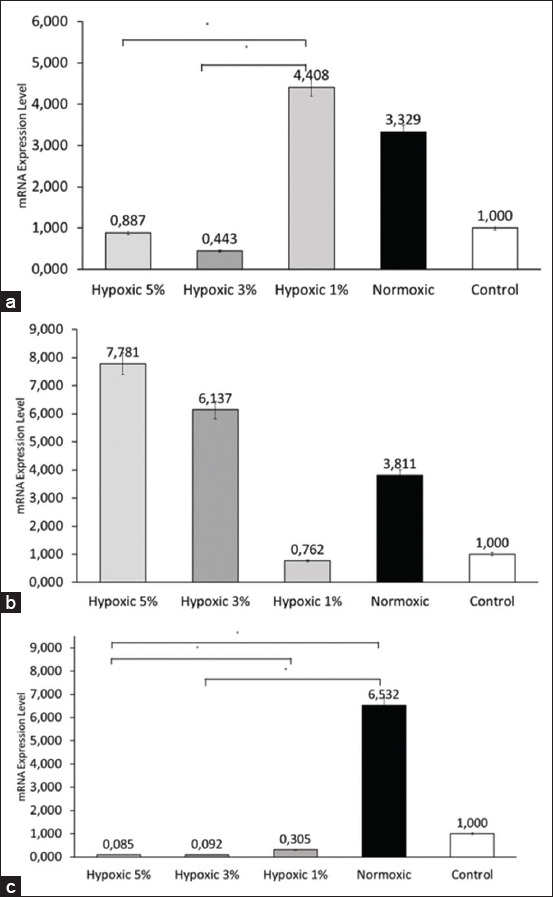
Angiogenesis gene expression profiles. (a) The mRNA expression level of *VEGF* in NHP UC-MSCs treated with 1% hypoxia was higher than that in the other groups. Significant differences were observed (p < 0.05) between the 1% and 3% hypoxic groups and the 1% and 5% hypoxic groups, marked with the line bars with asterisks. Non-significant differences were observed between the normoxic and 1% groups (p = 0.156), 3% and 5% groups (p = 0.997), normoxic and 3% groups (p = 0.117), and normoxic and 5% groups (p = 0.195). (b) The mRNA expression level of *MCP-1* from NHP UC-MSC with 5% hypoxic treatment was higher than in other groups. Non-significant differences were found between all groups (p = 0.134). (c) Histogram of *MMP-2* mRNA expression level between groups. Normoxic NHP UC-MSC exhibited the highest mRNA expression level of *MMP-2*. Significant differences were observed (p < 0.05) between the normoxic and 3% hypoxic groups, normoxic and 5% hypoxic groups, and 1% and 5% hypoxic groups, as marked with the line bars with asterisks. Non-significant differences were found between the normoxic and 1% hypoxic groups (p = 0.200), 1% and 3% hypoxic groups (p = 0.343), and 3% and 5% hypoxic groups (p = 0.686). All values are expressed as means with error bars indicating the standard deviation. mRNA=MicroRNA, *VEGF=*Vascular endothelial growth factor, UC-MSCs=Cord derived-mesenchymal stem cell, *MCP=*Monocyte chemoattractant protein, *MMP=*Matrix metalloproteinase, NHP=Non-human primates.

Our study found that in the VEGF groups, the oxygen concentration in the 1% group was more upregulated than that in the normoxic group. However, the expression of *VEGF* was downregulated in the other hypoxic groups, with the 3% group showing the most downregulation (Figure 1a). We also found that *MCP-1* gene expression was upregulated in most hypoxic groups, except in the 1% oxygen concentration group. The relative mRNA expression levels in the 3% and 5% oxygen concentration groups were higher than those in the normoxic group, where the 5% concentration group exhibited the highest upregulation (Figure 1b).

The hypoxic preconditioning used in this study resulted in the downregulation of *MMP-2* gene expression, whereas *MMP-2* was significantly upregulated under normoxic conditions. However, in the context of hypoxic preconditioning, we also found that *MMP-2* expression level increased gradually from the highest concentration to the lowest concentration, with the 1% concentration group being the most upregulated compared with the other hypoxic groups, as shown in Figure 1c.

### *miRNA-128-5p* expression profile

**Figure 2:**
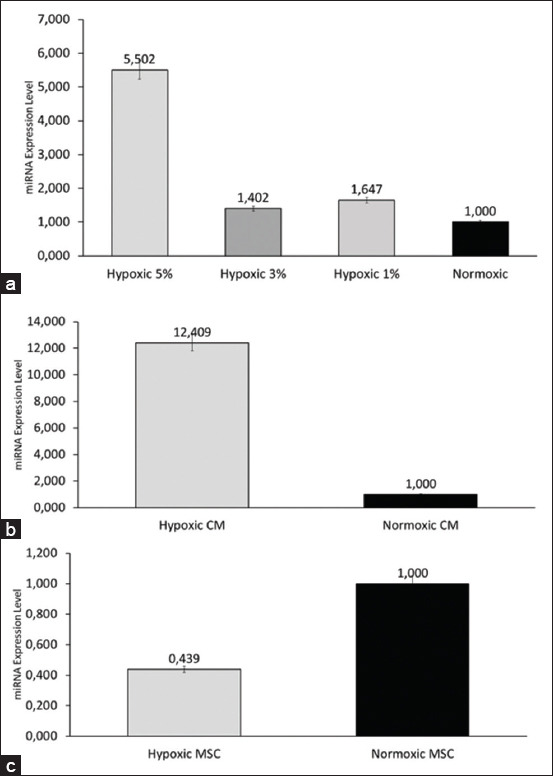
Epigenetics *miRNA-128* expression profiles. (a) miRNA expression levels between different preconditioning groups. Hypoxic preconditioning induces upregulation of *miRNA-128-5p*. The 5% hypoxic group had the highest *miRNA-128-5p* expression. Non-significant differences were found between groups (p = 0.814). (b) *miRNA-128-5p* expression level in the CM hypoxic group was higher than the normoxic group. Non-significant differences were found between secretome groups (p = 0.219). (c) *miRNA-128-5p* expression level in the UC-MSC hypoxic group was lower than the normoxic group. Non-significant differences were found between MSC groups (p = 0.286). All values are expressed as means with error bars indicating the standard deviation. miRNA=MicroRNA, UC-MSCs=Cord derived-mesenchymal stem cell, CM=Conditioned medium.

This study showed that hypoxic preconditioning caused upregulation of *miRNA-128-5p* expression in all hypoxic groups. Compared with the other preconditioning groups, the 5% group was the most upregulated, with 5-fold expression level changes compared with the normoxic group, whereas the 3% oxygen concentration group was the least regulated (Figure 2a).

In this study, we also evaluated differences in *miRNA-128-5p* expression between different cellular compartments. As depicted in Figure 2b, when exposed to a hypoxic microenvironment, *miRNA-128-5p* was found to be upregulated 12-fold in the secretome. Interestingly, we also found contrasting results (Figure 2c) regarding the expression of *miRNA-128-5p* in the hypoxic MSCs, which was lower than that in the normoxic group.

## DISCUSSION

In this study, we used archived samples prepared in our previous study, which were collected from the fetal tissues of long-tailed monkeys. The preconditioning stage was conducted for 48 h to increase the expression of hypoxia-inducible factor-1α (HIF-1α) while also avoiding cell death, as previously reported by Lee *et al*. [22]. VEGF is a protein family comprising various isoforms that play a role in the regulation of angiogenesis [4]. In this study, we found that both normoxic condition and hypoxic preconditioning with a 1% oxygen level could lead to VEGF upregulation, whereas the 1% preconditioning group showed increased VEGF upregulation. Another study also showed that a preconditioning process using 1% oxygen could lead to greater VEGF-A expression than normoxic conditions [23]. Low oxygen concentration leads to the stabilization of HIF-1α and subsequently induces VEGF as its primary target gene [24, 25].

MCP-1 is a type of chemokine that can regulate the migration and infiltration of immune cells as well as stem cells [26, 27]. Although we showed that the hypoxic groups were more upregulated than the normoxic group, we also found that a lower oxygen concentration could also lower the mRNA expression of *MCP-1*. This phenomenon may be caused by a decrease in the activity of oxygen-dependent enzymes, such as Jumonji C domain-containing histone demethylases, under chronic hypoxic conditions that lead to methylation in the promoter and enhancer regions of the *MCP-1* gene [28]. Overall, we found that 5% oxygen was the optimum oxygen concentration to induce *MCP-1* expression.

MMP-2 is a proteolytic enzyme with various biological activities [29, 30]. In this study, we found that the expression of *MMP-2* was upregulated in the normoxic group compared with the control group. We also found that the expression of *MMP-2* was downregulated in all hypoxic groups compared with the control group. To date, there have been no reports on the expression of *MMP-2* in MSCs subjected to hypoxic preconditioning. Findings from Ben-Yosef *et al*. [31] indicate that exposure to hypoxia conditions for 6 and 24 h can result in the downregulation of *MMP-2* in endothelial cells. MMP-2 also exhibits a dual role, as excessive release of this enzyme can compromise the integrity of the blood-brain barrier, potentially leading to hemorrhage [32]. Consequently, it is crucial to maintain *MMP-2* expression within a safe range to prevent harmful effects in the developed secretome. In addition, among the three concentrations examined under hypoxic conditions, lower oxygen levels were correlated with increased *MMP-2* mRNA expression. Therefore, optimization of *MMP-2* expression induction in cell culture is necessary to identify the optimal combination of time and oxygen concentration for optimal *MMP-2* expression.

Lanza *et al*. [12] have shown that aberrant *miRNA-128* expression can lead to pathological conditions or neuronal cell death. In this study, we found that hypoxia caused upregulation of *miRNA-128-5p* compared with the control. We also found another study reporting that a hypoxic condition during ischemic events could upregulate *miRNA-128* [33]. Furthermore, we also compared *miRNA-128* expression in different compartments under normoxic and hypoxic conditions. We found that during the normoxic condition, *miRNA-128-5p* is widely expressed in cells under normoxic conditions, whereas *miRNA-128-5p* is widely secreted to the secretome under hypoxic conditions. The release of miRNA into the extracellular space is believed to be affected by certain pathways. Because we cultured the cells in hypoxia, we hypothesized that a hypoxic condition might play a role in modulating the expression of *miRNA-128* through mechanisms such as HIF upregulation, epigenetic modifications, and the modulation of proteins involved in the maturation of miRNA [34]. After this modulation, these excess miRNAs can then be secreted outside the cells and play various functional roles, including in intercellular communication [35]. However, further research is required to investigate the exact function of miRNA release outside the cells. Overall, we found that hypoxic preconditioning can upregulate *miRNA-128-5p* and induce its release from the cell to the secretome.

Our findings pave the way for optimizing stem cell culture conditions that can induce the expression of some biomarkers that have a role in the process of angiogenesis and anti-apoptosis as the first step to characterize bioactive components present in the secretome of UC-MSCs. The characterization process is essential for ensuring safety and compliance with regulatory standards [36, 37], promoting the standardization and reproducibility of the secretome [38], and aiding in elucidating the role of each bioactive component in the regenerative effects, thereby facilitating the clinical translation process [1, 36]. In conclusion, different cell culture conditions could influence various gene expressions involved in the angiogenesis process. This study suggests that hypoxic UC-MSCs and secretomes from NHP are potential candidates for stroke therapy.

## CONCLUSION

This study highlights the significant influence of oxygen concentration on the expression of key angiogenic and anti-apoptotic factors in MSC cultures. Hypoxic preconditioning at 5% oxygen effectively upregulated *MCP-1* and *miRNA-128*, while 1% oxygen maximized *VEGF* expression. In contrast, normoxic conditions (21% oxygen) were optimal for *MMP-2* expression, suggesting that specific oxygen levels can selectively modulate gene expression to enhance therapeutic potential. In addition, *miRNA-128* was predominantly secreted into the extracellular space under hypoxia, emphasizing its potential role in paracrine signaling.

These findings underscore the therapeutic potential of hypoxia-conditioned UC-MSCs and their secretomes as a promising regenerative approach for stroke therapy and other ischemic conditions. The use of non-human primate models (*M. fascicularis*) further supports the translational relevance of these findings.

Future research should focus on *in vivo* validation to assess the efficacy of hypoxia-conditioned UC-MSCs and their secretomes in preclinical stroke models. Further investigations are also required to determine the optimal duration of hypoxic exposure, the stability of secretome-derived bioactive factors, and the downstream molecular pathways regulated by miRNA-128. Expanding this research to other neurodegenerative and ischemic disease models will further validate the clinical applicability of hypoxic preconditioning strategies for MSC-based therapies.

## DATA AVAILABILITY

The raw data supporting the conclusions of this article will be made available from the corresponding author upon a reasonable request.

## AUTHORS’ CONTRIBUTIONS

HANU, SSM, AD, RR, and AM: Designed and performed the study, analyzed data, and drafted the manuscript. HANU, SSM, RR, AD, YRP, PRI, GIR, HHI, WA, RN, FH, AF, SS, and AM: Validated the methodology and tests. HANU, SSM, and AM: Drafted the manuscript. SSM, RR, and AM: Supervised the study. All authors have read and approved the final manuscript.

## References

[1] Drago D, Cossetti C, Iraci N, Gaude E, Musco G, Bachi A, Pluchino S (2013). The stem cell secretome and its role in brain repair. Biochimie.

[2] Xia J, Minamino S, Kuwabara K, Arai S (2019). Stem cell secretome as a new booster for regenerative medicine. Biosci. Trends.

[3] Pawitan J.A (2014). Prospect of stem cell conditioned medium in regenerative medicine. Biomed. Res. Int 2014.

[4] Apte R.S, Chen D.S, Ferrara N (2019). VEGF in signaling and disease:Beyond discovery and development. Cell.

[5] Ruan L, Wang B, Zhuge Q, Jin K (2015). Coupling of neurogenesis and angiogenesis after ischemic stroke. Brain Res.

[6] Deshmane S.L, Kremlev S, Amini S, Sawaya B.E (2009). Monocyte chemoattractant protein-1 (MCP-1):An overview. J. Interf. Cytokine Res.

[7] Yan Y.P, Sailor K.A, Lang B.T, Park S.W, Vemuganti R, Dempsey R.J (2007). Monocyte chemoattractant protein-1 plays a critical role in neuroblast migration after focal cerebral ischemia. J. Cereb. Blood Flow Metab.

[8] Masuda T, Isobe Y, Aihara N, Furuyama F, Misumi S, Kim T.S, Nishino H, Hida H (2007). Increase in neurogenesis and neuroblast migration after a small intracerebral hemorrhage in rats. Neurosci. Lett.

[9] Morancho A, Rosell A, García-Bonilla L, Montaner J (2010). Metalloproteinase and stroke infarct size:Role for anti-inflammatory treatment?. Ann. N. Y. Acad. Sci.

[10] Uzdensky A.B (2019). Apoptosis regulation in the penumbra after ischemic stroke:Expression of pro-and antiapoptotic proteins. Apoptosis.

[11] O'Brien J, Hayder H, Zayed Y, Peng C (2018). Overview of microRNA biogenesis, mechanisms of actions, and circulation. *Front. Endocrinol*. (Lausanne).

[12] Lanza M, Cuzzocrea S, Oddo S, Esposito E, Casili G (2023). The role of miR-128 in neurodegenerative diseases. Int. J. Mol. Sci.

[13] Wan X, Yao B, Ma Y, Liu Y, Tang Y, Hu J, Li M, Fu S, Zheng X, Yin D (2020). MicroRNA-128-1-5p attenuates myocardial ischemia/reperfusion injury by suppressing Gadd45g-mediated apoptotic signaling. Biochem. Biophys. Res. Commun.

[14] Dumingan A, Malik A, Rinendyaputri R, Utama H.A.N, Sunarno Purwaningtyas Y.R, Idrus H.H, Noverina R, Huda F, Faried A (2024). Characteristics of umbilical cord derived mesenchymal stem cell/UCMSC from *Macaca fascicularis* and its secretome under hypoxic conditions. Biota:Biol. Dan Pendidik. Biol.

[15] Meunier J, Lemoine F, Soumillon M, Liechti A, Weier M, Guschanski K, Hu H, Khaitovich P, Kaessmann H (2013). Birth and expression evolution of mammalian microRNA genes. Genome Res.

[16] Jin L.S, Rao J.H, Zhang L.B, Ji F, Zhang Y.C, Hao X.F, Peng B.L, Liu X.M, Sun Y.X (2019). Comparison of gene expression in cynomolgus monkeys with preclinical type II diabetes induced by different high energy diets. Animal Model Exp. Med.

[17] Mariya S.S, Dewi F.N, Villiandra V, Pramastri Y.A, Iskandriati D, Hayes E, Pamungkas J, Lelana R.A, Tumbelaka L.I, Sajuthi D (2018). CCL2 and CCR2 expression in broncoalveolar lavage fluid of cynomolgus macaque model of asthma. J. Respirol. Indones.

[18] Khalil C, Moussa M, Azar A, Tawk J, Habbouche J, Salameh R, Ibrahim A, Alaaeddine N (2019). Anti-proliferative effects of mesenchymal stem cells (MSCs) derived from multiple sources on ovarian cancer cell lines:An *in-vitro* experimental study. J. Ovarian Res.

[19] Ge L, Xun C, Li W, Jin S, Liu Z, Zhuo Y, Duan D, Hu Z, Chen P, Lu M (2021). Extracellular vesicles derived from hypoxia-preconditioned olfactory mucosa mesenchymal stem cells enhance angiogenesis via miR-612. J. Nanobiotechnology.

[20] Rosmanah L, Saepuloh U, Mariya S.S, Suparto I.H, Manalu W, Winarto A, Darusman H.S (2024). Expression of APP, CDK5, and AKT1 gene related to Alzheimer disease in brain of long-tailed macaques. HAYATI J. Biosci.

[21] Pfaffl M.W (2001). A new mathematical model for relative quantification in real-time RT-PCR. Nucleic Acids Res.

[22] Lee S.M, Jun D.W, Kang H.T, Oh J.H, Saeed W.K, Ahn S.B (2021). Optimal hypoxic preconditioning of human embryonic stem cell-derived mesenchymal stem cells (hES-MSCs) and their characteristics. Int. J. Stem Cells.

[23] Muzakkir A.F, Suryawan I.G.R, Yusrizal T (2020). Hypoxic preconditioning effects of bone marrow-derived culture mesenchymal stem cells on CD31+expression, vascular endothelial growth factor-a (VEGF-A) and stromal-derived factors-1 alpha (SDF-1). IOP Conf. Ser. Earth Environ. Sci.

[24] Ramakrishnan S, Anand V, Roy S (2014). Vascular endothelial growth factor signaling in hypoxia and inflammation. J. Neuroimmune Pharmacol.

[25] Moeinabadi-Bidgoli K, Babajani A, Yazdanpanah G, Farhadihosseinabadi B, Jamshidi E, Bahrami S, Niknejad H (2021). Translational insights into stem cell preconditioning:From molecular mechanisms to preclinical applications. Biomed. Pharmacother.

[26] Singh S, Anshita D, Ravichandiran V (2021). MCP-1:Function, regulation, and involvement in disease. Int. Immunopharmacol.

[27] Widera D, Holtkamp W, Entschladen F, Niggemann B, Zänker K, Kaltschmidt B, Kaltschmidt C (2004). MCP-1 induces migration of adult neural stem cells. Eur. J. Cell Biol.

[28] Korbecki J, Kojder K, Barczak K, Simińska D, Gutowska I, Chlubek D, Baranowska-Bosiacka I (2020). Hypoxia alters the expression of CC chemokines and CC chemokine receptors in a tumor-a literature review. Int. J. Mol. Sci.

[29] Laronha H, Caldeira J (2020). Structure and function of human cells. Cells.

[30] Almalki S.G, Agrawal D.K (2016). Effects of matrix metalloproteinases on the fate of mesenchymal stem cells. Stem Cell Res. Ther.

[31] Ben-Yosef Y, Lahat N, Shapiro S, Bitterman H, Miller A (2002). Regulation of endothelial matrix metalloproteinase-2 by hypoxia/reoxygenation. Circ. Res.

[32] Lakhan S.E, Kirchgessner A, Tepper D, Leonard A (2013). Matrix metalloproteinases and blood-brain barrier disruption in acute ischemic stroke. Front. Neurol.

[33] Liu P, Han Z, Ma Q, Liu T, Wang R, Tao Z, Li G, Li F, Zhang S, Li L, Ji X, Zhao H, Luo Y (2019). Upregulation of MicroRNA-128 in the peripheral blood of acute ischemic stroke patients is correlated with stroke severity partially through inhibition of neuronal cell cycle reentry. Cell Transplant.

[34] Nallamshetty S, Chan S.Y, Loscalzo J (2013). Hypoxia:A master regulator of microRNA biogenesis and activity. Free Radic. Biol. Med.

[35] Zhao C, Sun X, Li L (2019). Biogenesis and function of extracellular miRNAs. ExRNA.

[36] Gwam C, Mohammed N, Ma X (2021). Stem cell secretome, regeneration, and clinical translation:A narrative review. Ann. Transl. Med.

[37] Kim J.H, Green D.S, Ju Y.M, Harrison M, Vaughan J.W, Atala A, Lee S.J, Jackson J.D, Nykiforuk C, Yoo J.J (2022). Identification and characterization of stem cell secretome-based recombinant proteins for wound healing applications. Front. Bioeng. Biotechnol.

[38] Chouaib B, Haack-Sørensen M, Chaubron F, Cuisinier F (2023). Towards the standardization of mesenchymal stem cell secretome-derived product manufacturing for tissue regeneration. Int. J. Mol. Sci.

